# Biomolecules as Model Indicators of *In Vitro* and *In Vivo* Cold Plasma Safety

**DOI:** 10.3389/fphy.2020.613046

**Published:** 2021-01-14

**Authors:** Caitlin Heslin, Daniela Boehm, Brendan F. Gilmore, Julianne Megaw, Theresa A. Freeman, Noreen J. Hickok, P. J. Cullen, Paula Bourke

**Affiliations:** 1 School of Food Science and Environmental Health, Technological University Dublin, Dublin, Ireland; 2 School of Pharmacy, Queens University Belfast, Belfast, United Kingdom; 3 Department of Orthopaedic Surgery, Sidney Kimmel Medical College, Jefferson University, Philadelphia, PA, United States; 4 School of Chemical and Biomolecular Engineering, University of Sydney, Sydney, NSW, Australia; 5 School of Biological Sciences, Queens University Belfast, Belfast, United Kingdom; 6 Plasma Research Group, School of Biosystems and Food Engineering, University College Dublin, Dublin, Ireland

**Keywords:** cold atmospheric plasma, cytotoxicity, mutagenicity, safety, *In vivo* toxicity

## Abstract

The potential applications for cold plasma in medicine are extensive, from microbial inactivation and induction of apoptosis in cancer cells to stimulating wound healing and enhancing the blood coagulation cascade. The safe bio-medical application of cold plasma and subsequent effect on complex biological pathways requires precision and a distinct understanding of how physiological redox chemistry is manipulated. Chemical modification of biomolecules such as carbohydrates, proteins, and lipids treated with cold plasma have been characterized, however, the context of how alterations of these molecules affect cell behavior or *in vivo* functionality has not been determined. Thus, this study examines the cytotoxic and mutagenic effects of plasma-treated molecules *in vitro* using CHO-K1 cells and *in vivo* in *Galleria mellonella* larvae. Specifically, albumin, glucose, cholesterol, and arachidonic acid were chosen as representative biomolecules, with established involvement in diverse bioprocesses including; cellular respiration, intracellular transport, cell signaling or membrane structure. Long- and short-term effects depended strongly on the molecule type and the treatment milieu indicating the impact of chemical and physical modifications on downstream biological pathways. Importantly, absence of short-term toxicity did not always correlate with absence of longer-term effects, indicating the need to comprehensively assess ongoing effects for diverse biological applications.

## INTRODUCTION

The biological effects of cold plasma are complex and occur at the biological interface between biophysics, biochemistry and cell biology. Cold plasma is produced by applying energy to a gaseous environment. As the gas is ionized a complex mixture of reactive components is generated. The application of cold plasma technology to biological targets has revealed effectiveness in a diverse range of activities from promoting cell proliferation [1], blood coagulation [2], cancer treatment [3, 4], and inducement of specific cell senescence [5]. Other noteworthy applications of cold plasma have included disinfection potentially with reduced risk of antimicrobial resistance [6–8], decontamination of fresh produce [9], bio-decontamination of heat sensitive products [10], seeds and grains with an aim for human consumption [11, 12] and areas key to the sustainability of food and agriculture [13, 14].

The biological effects of plasma and plasma generated chemistry are dependent on the surrounding liquid environment [15–18]. In fact, studies have indicated immense variability in the biological effects attributed to cold plasma, depending on both the composition of the sample substrate and that of the ionized gas. Thus, for successful application of cold plasma technology it is important to not only understand interactions at the bio-plasma interface during treatment, but also to understand the short-term and long-term consequences of plasma induced biomolecule alteration and the effect this may have on biochemical processes. In fact, plasma induced chemical modification of proteins and amino acids [19, 20] and lipids [21] have been studied in isolation. Additionally, both simulated, modeling studies and direct experimentation have shown how interactions of cold plasma chemical species modify amino acids [22, 23], change protein structure and function [19, 24–26] and oxidize lipids [27, 28] but these studies are limited in their ability to examine or understand the long-term biological effects of the plasma induced modifications in a complex biosystem.

Encouragingly, investigations of direct plasma jet [29] or surface micro-discharge treatment on mammalian cells showed negligible mutagenic effects [30] and no mutagenic potential using the *in vivo* HET-MN model [31]. In fact, a range of investigations on human or animal tissues *in vivo* and clinical application of several certified plasma devices suggest that cold plasma treatment under these conditions is well tolerated and safe [32] and no long-term adverse effects have been reported to-date [33, 34]. However, other studies have also demonstrated that cold plasma can be modulated to become a powerful mutagenesis tool under appropriate conditions [35, 36] and mutagenic effects of cold plasma treated complex biofluids on mammalian cells have been documented, but not directly elucidated [37], suggesting that genetic damage is possible where cells experience exposure to intense plasma or plasma reactive species over extensive periods of time.

In an effort to reconcile these differences, this study examines the cytotoxic and mutagenic potential of plasma-treated biomolecules using both *in vitro* and *in vivo* models chosen for relevance to a range of biological environments which incorporate protein, lipid and carbohydrate components. Bovine serum albumin was selected as a representative protein molecule and for its chemical similarity to human serum albumin which is an abundant antioxidant protein in the blood. Arachidonic acid was chosen as an essential omega-6 polyunsaturated fatty acid [38] with four cis double bonds that contributes to mammalian cell membrane fluidity at physiological temperatures and is a precursor of eicosanoids [39]. Cholesterol, a sterol synthesised by all animal cells, is required for membrane structural integrity and flexibility. Cholesterol is a monounsaturated fatty acid precursor for all steroid hormones and functions in intracellular transport and cell signaling with the formation of lipid rafts in the plasma membrane [40]. Glucose was selected as a model carbohydrate and ubiquitous fuel source in biology. The safe application of cold plasma and subsequent effect on complex biochemical pathways requires precision and a distinct understanding of how physiological redox chemistry is manipulated.

## RESULTS

### Cytotoxicity Dependent on Biomolecular Structure

**FIGURE 1 |:**
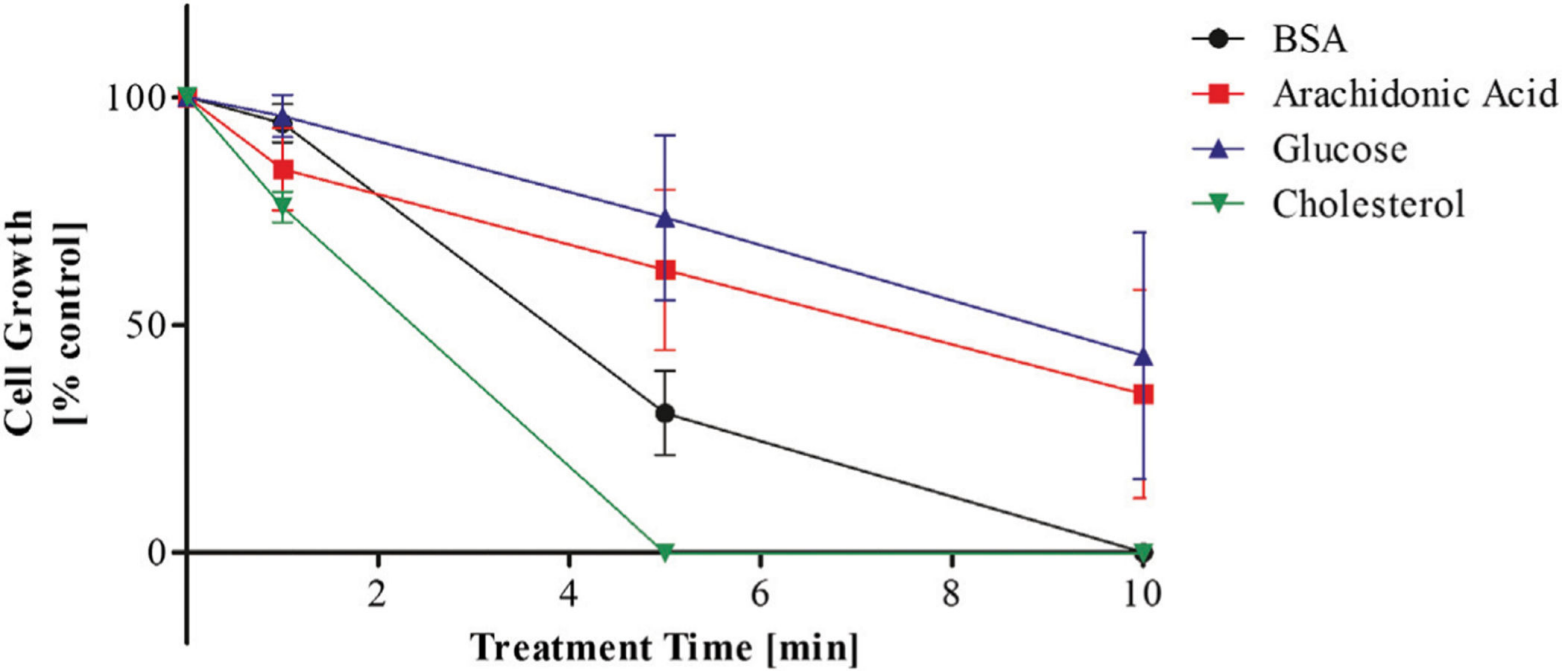
Cytotoxicity of plasma treated biomolecules dissolved in deionised water over plasma treatment time.

To assess the short-term cytotoxic effect of plasma treated biomolecules, selected biomolecules were dissolved in deionised water and subjected to plasma treatment. CHO-K1 cells were seeded at 2.5 ×10^4^ cells/ml with 20% v/v of these treated biomolecules and cell growth was assessed after 2–3 days (Figure 1).

Prolonged plasma treatment of the bio-molecular solutions induced cytotoxicity in the CHO-K1 cell line, which was dependent on plasma treatment time (Figure 1). Plasma treated cholesterol elicited the strongest cytotoxic reaction. The growth of CHO-K1 cells cultured with plasma treated cholesterol was reduced to 75% with 1 min of plasma treatment compared to 94% (BSA), 84% (arachidonic acid), and 96% (glucose) and was less than 1% when cultured with cholesterol treated for 5 and 10 min. This is in contrast to arachidonic acid and glucose, where cell growth was reduced to 62% and 74% with 5 min treatment and decreased to 35% and 43% with 10 min of treatment respectively. CHO-K1 cells cultured with BSA plasma treated for 5 min were reduced to 30% and failed to grow when cultured with BSA treated for 10 min.

**FIGURE 2 |:**
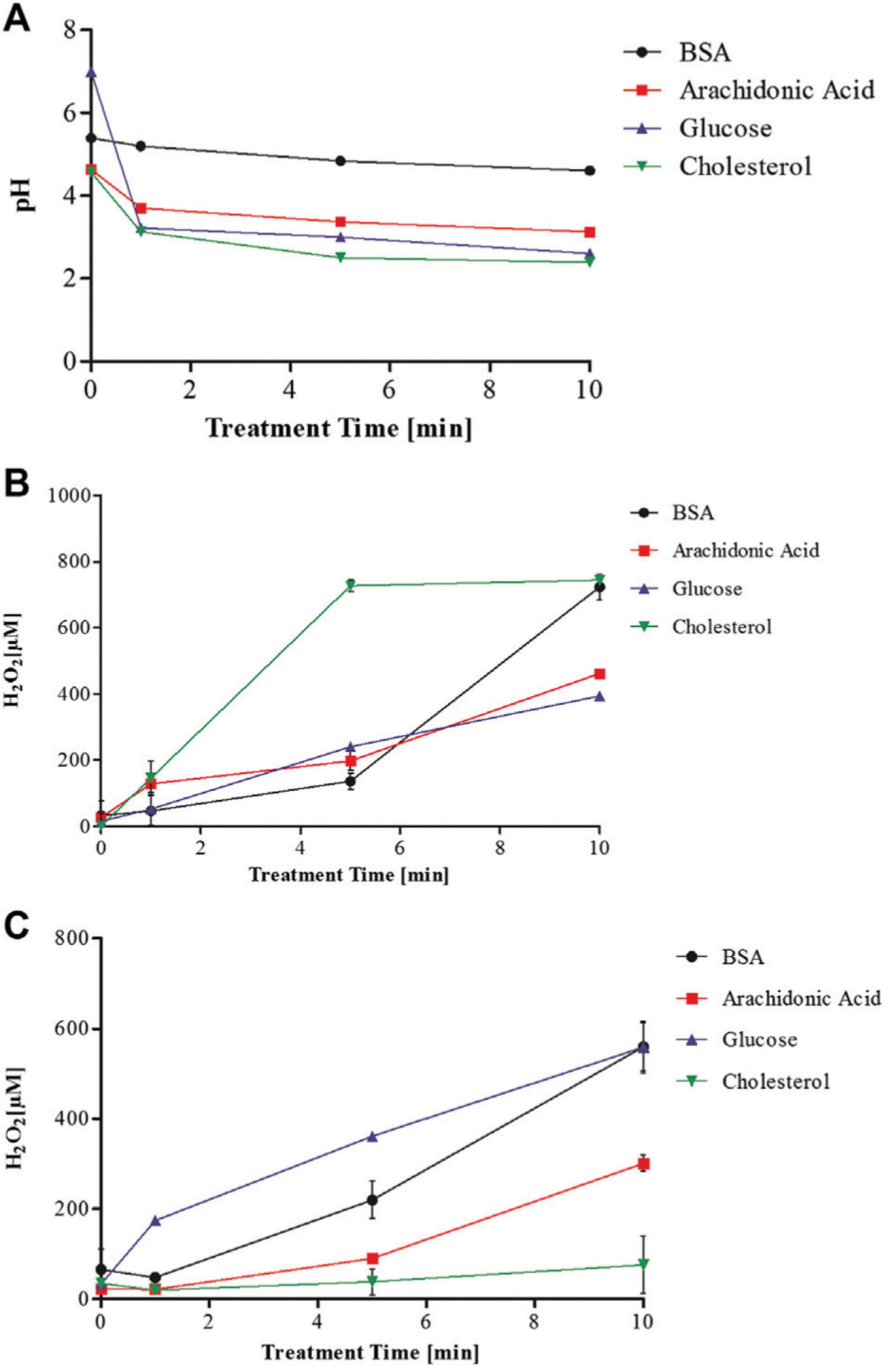
pH of plasma treated biomolecules dissolved in deionized water **(A)** and hydrogen peroxide quantification of plasma treated biomolecules dissolved in deionised water **(B)** and dissolved in DMEM-F12 **(C)**.

**FIGURE 3 |:**
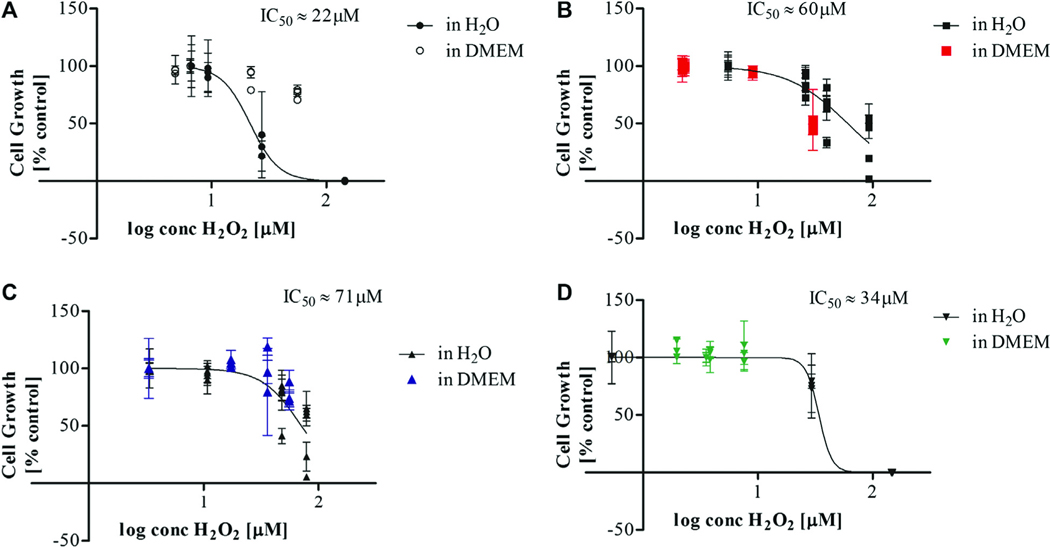
IC50 determination for plasma treated biomolecules dissolved in deionised water and DMEM **(A)** BSA, **(B)** Arachidonic acid, **(C)** Glucose, **(D)** Cholesterol.

Exposing aqueous solutions to cold plasma treatment leads to the generation of hydrogen peroxide among other molecules, which may be a useful indicator of plasma activity and potential predictor of the cytotoxicity of the solutions. The aqueous treatment environment provides the substrate to generate complex plasma-liquid chemistry without the same quenching effect that cell culture media provides. Measurements of pH and hydrogen peroxide were used as an indicator of plasma-liquid chemistry (Figure 2). The H_2_O_2_ quantification of the plasma treated biomolecular solutions indicated that the surrounding media influences the generated plasma chemistry and may have an effect on the biomolecules or generation of secondary products (Figures 2B,C). As hydrogen peroxide has been identified as a major contributor to cytotoxic effects of plasma treated liquids, cell growth was correlated to the hydrogen peroxide concentration of the respective biomolecule solution used at 20% v/v and the IC50 was determined. Cell growth data obtained from biomolecules dissolved in DMEM-F12 and used at 10% v/v for the mutagenicity assays below, are plotted in the same graphs for comparison purposes (Figure 3).

A comparison of the plasma-treated biomolecule solutions as a function of their hydrogen peroxide content, serves to indicate whether the differences in cytotoxicity are based on differences in their scavenging of hydrogen peroxide or on toxic modifications of the biomolecules themselves. The IC50 values of hydrogen peroxide for plasma treated biomolecules dissolved in deionised water ranged from 22 μM for BSA (Figure 3A), 34 μM for cholesterol (Figure 3B), to 60 μM for arachidonic acid (Figure 3C) and 71 μM for glucose solutions (Figure 3D). These data suggested that differences in hydrogen peroxide concentration were not the primary determinant of differences in toxicity. The dose-response curves obtained for biomolecules dissolved in DMEM displayed similar trends to those for biomolecules treated in water with the notable exception of BSA. The increased cytotoxicity associated with plasma treated BSA-water solutions may be due to the lack of pH buffering capacity accompanied by the more oxidative environment of the water with less scavenging potential for plasma generated reactive species. These conditions could potentially lead to the generation of toxic bi-products in the plasma treated aqueous biomolecular solutions that may not occur in the DMEM-F12 solution.

### Mutagenic Effect of Plasma Treated Biomolecules

**FIGURE 4 |:**
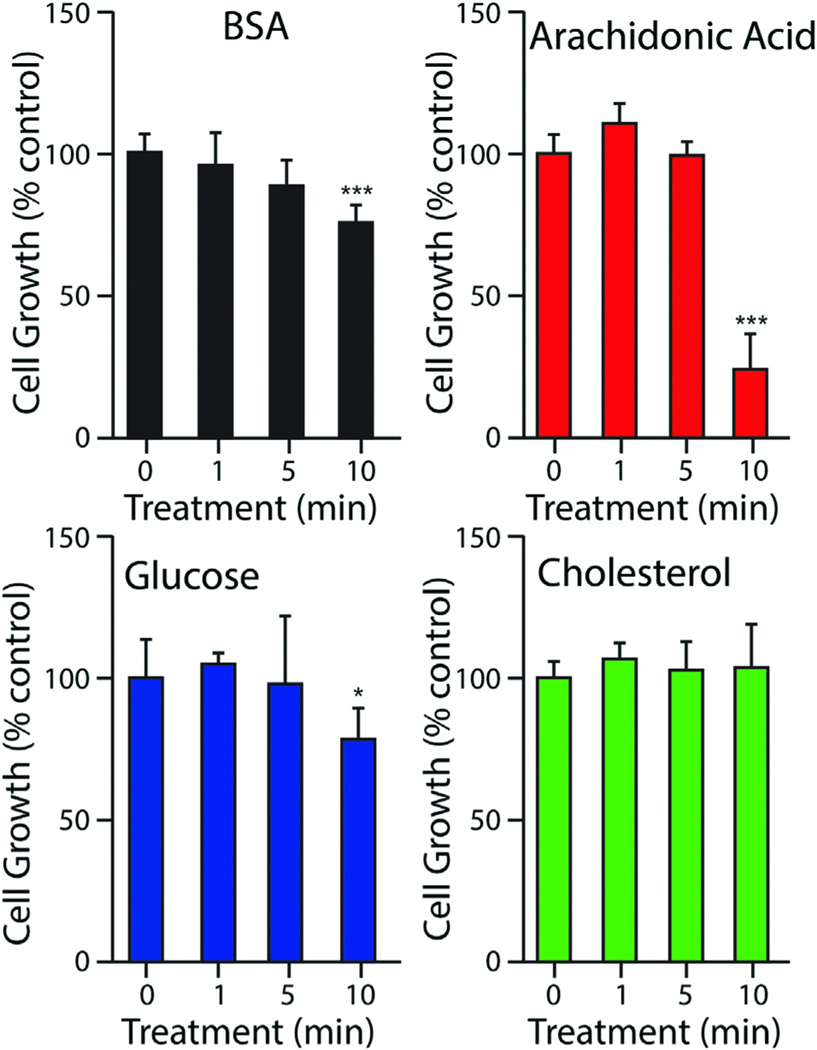
Growth of CHO-K1 cells in 10% v/v of physiological levels of selected biomolecules after plasma treatment. Different letters indicate a significant difference between treatment times (*p* < 0.05).

CHO-K1 cells were cultured with 10% v/v of biomolecules dissolved in DMEM-F12 to assess the long term mutagenic effects of plasma treated biomolecules using the hypoxanthine phosphoribosyl transferase (HPRT) assay. The growth of CHO-K1 cells cultured in 10% v/v of plasma treated solutions was assessed to ensure cells were growing in selected conditions (Figure 4). Cell growth of CHO-K1 cells cultured in arachidonic acid solution treated with plasma for 10 min was reduced by 50%. Cells that were cultured with plasma treated BSA and glucose were reduced to 75% and 78% respectively for the 10 min treated biomolecule (Figure 4). Plasma treated cholesterol did not appear to exhibit the same degree of cytotoxicity following prolonged plasma treatment as cell growth remained stable even at the extended treatment time of 10 min. This is in contrast to effects found for cholesterol treated in water above (Figure 3). The cytotoxic effects observed were independent of pH as the value of the buffered solutions did not decrease below a pH of 6.8 for the prolonged treatment time of 10 min for glucose and cholesterol and a pH of 7.1 for BSA and arachidonic acid.

**FIGURE 5 |:**
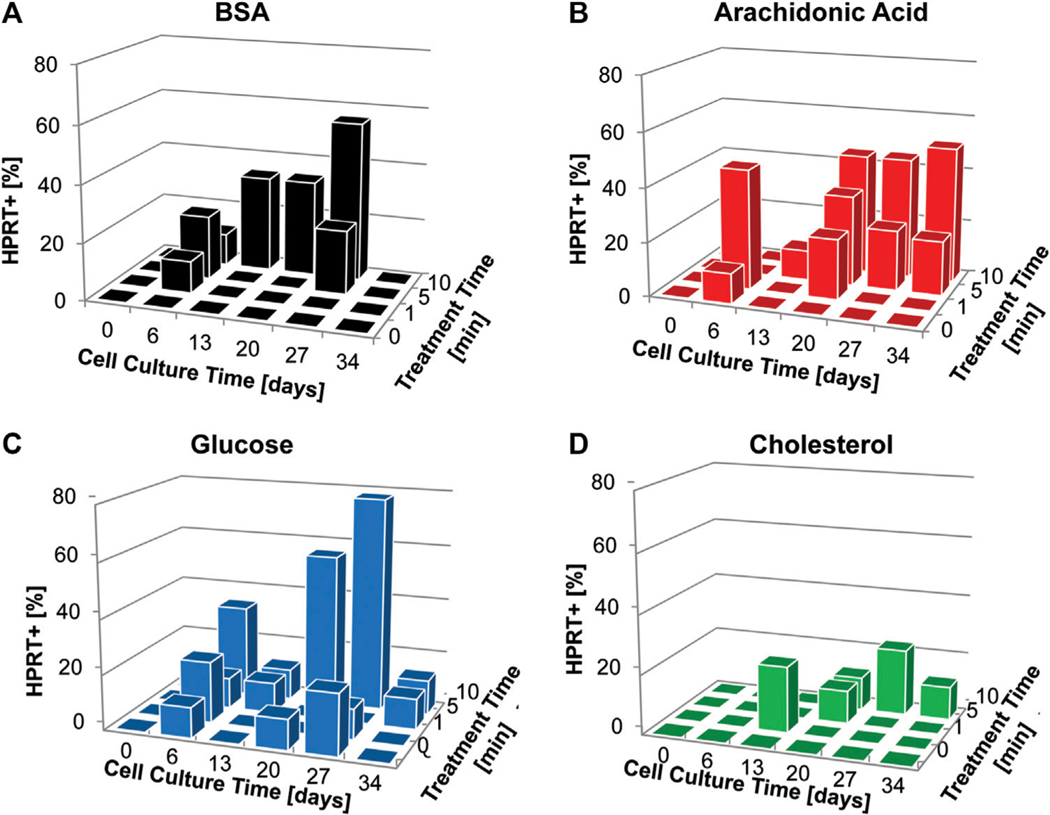
Colony formation (% HPRT+) for cultures supplemented with plasma treated biomolecules over the course of cell culture and according to plasma treatment **(A)** BSA, **(B)** Archidonic acid, **(C)** Glucose, **(D)** Cholesterol. Data is presented as cumulative data from experiments performed in triplicate, with all replicates plated in three independent plates at each time point. Plates were scored as positive or negative based on the presence of colony formation and are displayed as percentage positive plates of the overall plates assessed at each time point.

In order to assess the long-term mutagenic potential of plasma treated biomolecules, CHO-K1 cells were cultured with plasma treated biomolecule solutions over 34 days and monitored for HPRT-deficient mutants through colony formation in selective medium. Data is presented as cumulative data from experiments performed in triplicate, with all replicates plated in three independent plates at each time point and is displayed as percentage positive plates of the overall plates assessed at each time point (Original data available as Supplementary Table S1). Plasma treated biomolecules caused an increase in mutant colonies over time of culture and time of extended plasma treatment of the biomolecule solution (Figure 5).

HPRT colony formation in CHO-K1 cells cultured with plasma-treated BSA increased over cell culture period and with the prolonged plasma treatment time of 10 min (Figure 5A) and reached a maximum HPRT+ of 56% of replicates for 10 min plasma treatment after 27 days of exposure. Plasma treated arachidonic acid was consistently HPRT+ after 13 days of culture for 5 and 10 min plasma treatment times, with a maximum of 50% HPRT+ replicates on day 34 of culture (Figure 5B). Glucose displayed the greatest mutagenic potential aligned with plasma treatment time and cell culture period (Figure 5C). There was a maximum of 78% HPRT+ replicates for the 10 min plasma treated glucose after 27 days in culture. Plasma treated cholesterol exhibited low mutagenic potential even with the extended treatment time of 10 min reaching a maximum of 22% HPRT+ after 27 days in culture (Figure 5D).

**FIGURE 6 |:**
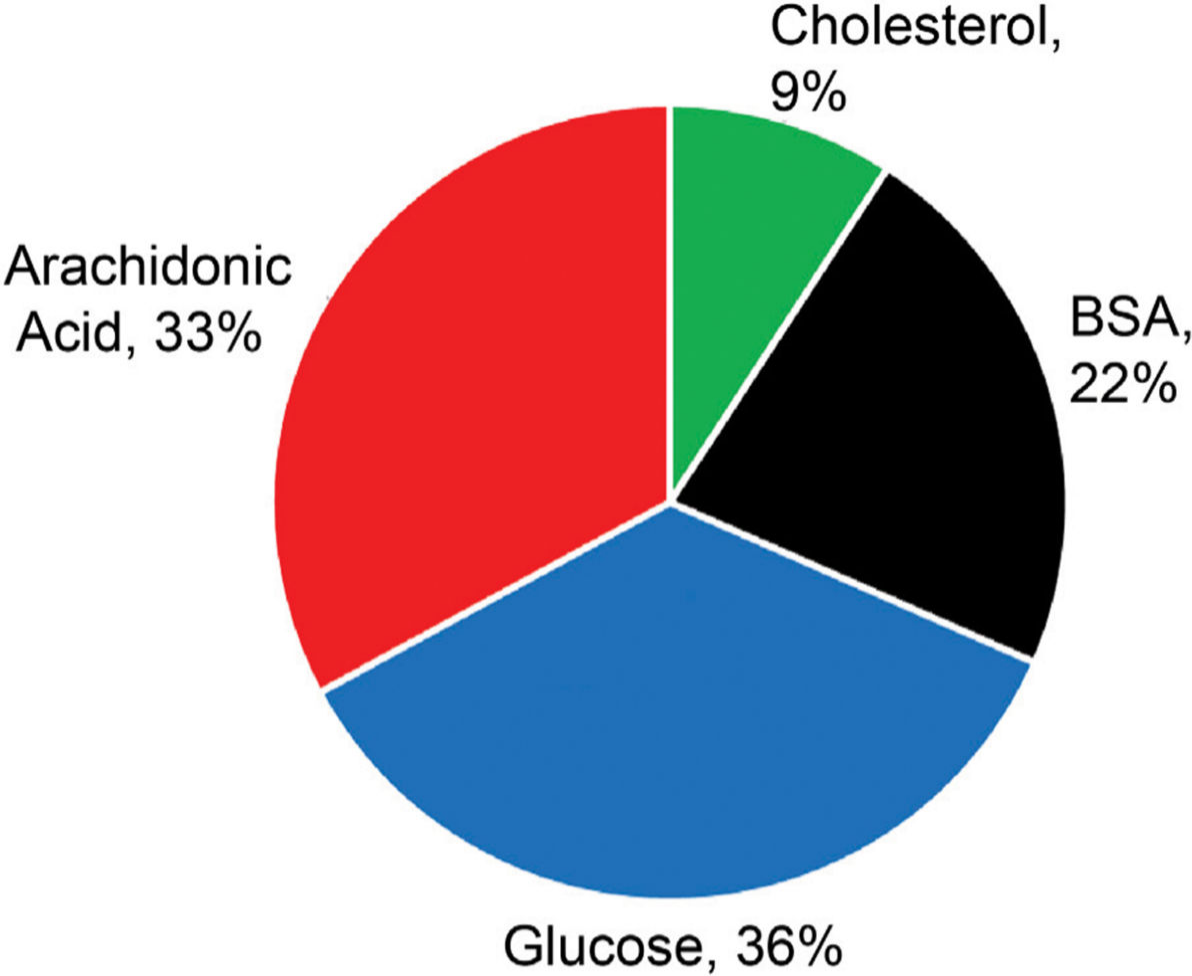
Colony formation (% total HPRT+) according to selected biomolecules.

Plasma treated glucose exhibited the greatest mutagenic potential accounting for 36% of the total HPRT+ colonies (Figure 6).

### *In vivo* Model System to Test Plasma Treated Biomolecule Toxicity

**FIGURE 7 |:**
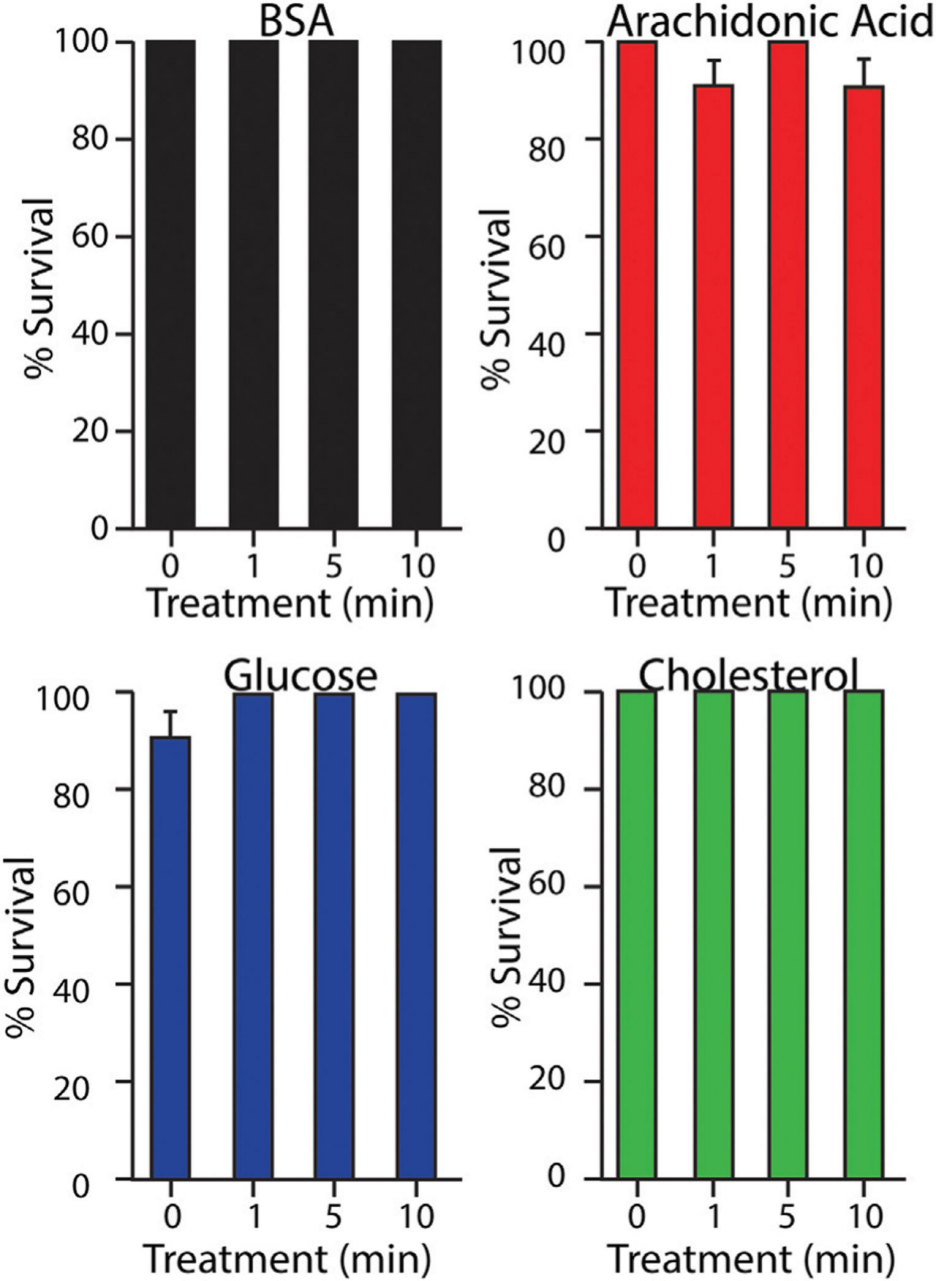
*Galleria mellonella* larvae survival rates 24 h post injection with plasma treated biomolecules.

Larvae of the wax moth *Galleria mellonella* were used as a model system to assess the toxicity of plasma treated biomolecules *in vivo* through injection. The plasma treated biomolecules were found to be well tolerated by the *Galleria* larvae with ∼100% survival (Figure 7). Haemocyte density was assessed as an indicator of overall larvae health. There was no significant increase in haemocyte density as would be observed if the larvae were under substantial stress (data not shown). For comparison, larvae were also injected with deionised water subjected to the same plasma treatment and hydrogen peroxide solutions at concentrations between 100 and 900 μM. The larvae tolerated the plasma treated deionised water well and haemocyte density was not affected (data not shown). The larvae did not tolerate the higher concentrations of hydrogen peroxide despite the concentrations being in the same range as measured in the plasma treated biomolecules (Figure 9). The surviving larvae were assessed for haemocyte density and levels were not significantly different from the control. This indicates that the immune system had not been challenged and that the solutions did not stimulate a non-selective immune response. Despite the equivalence in peroxide concentrations, this study indicated that there was no toxicity of the plasma treated biomolecules to the *in vivo* model *G. mellonella* under the conditions tested.

**FIGURE 8 |:**
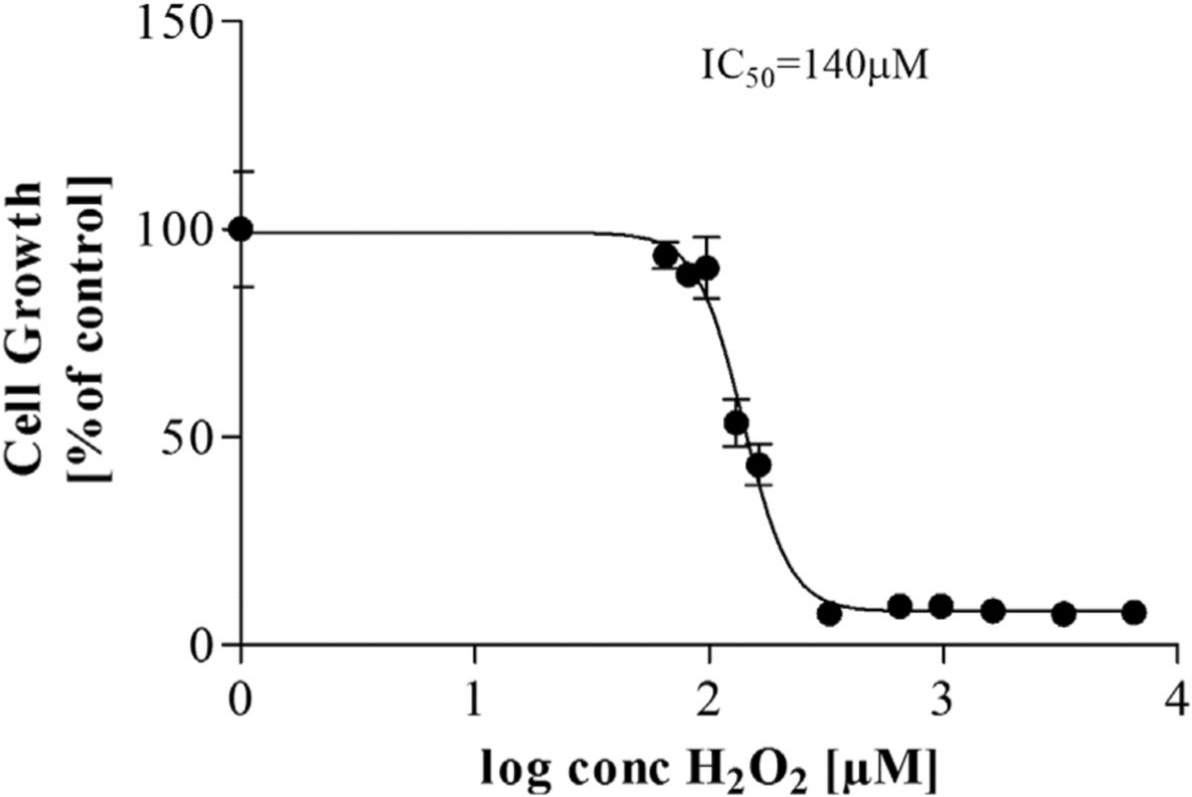
Dose-response curve for CHO-K1 cells treated with H_2_O_2_.

**FIGURE 9 |:**
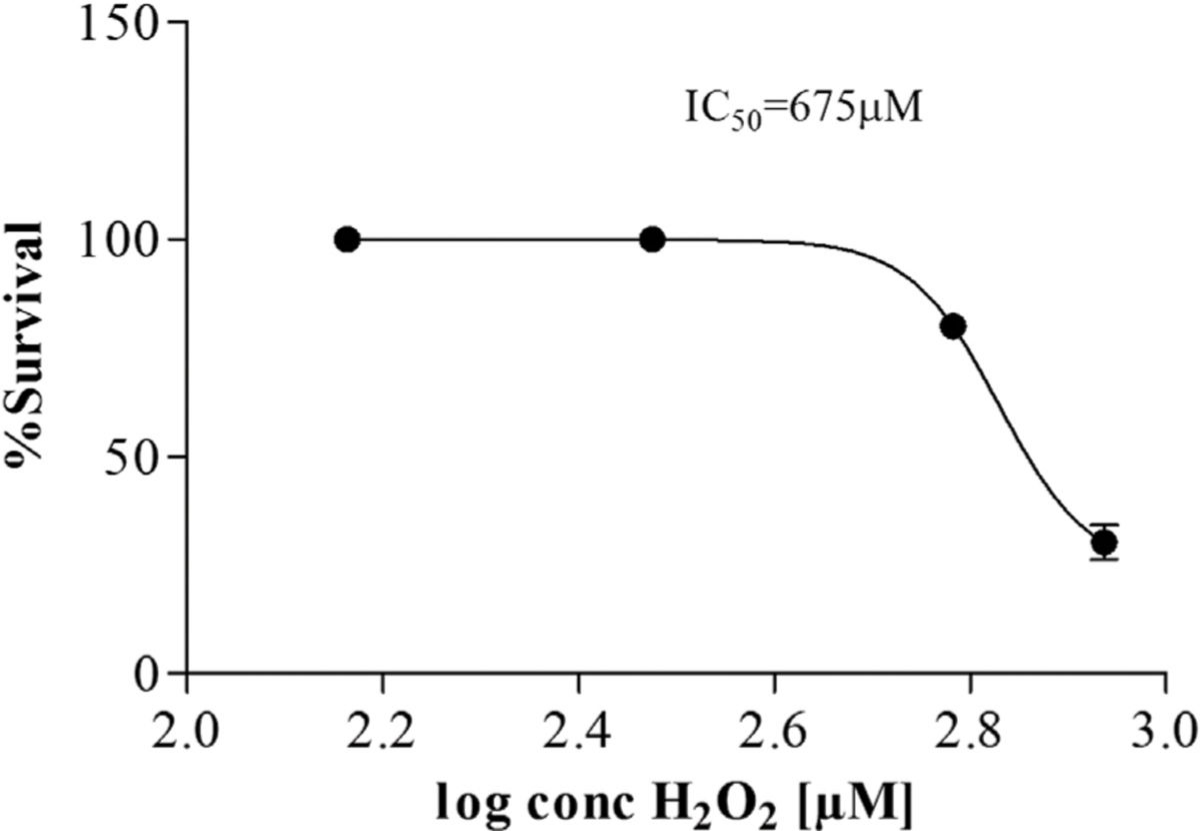
Dose-response curve for *Galleria mellonella* larvae injected with H_2_O_2_.

As a comparative indicator of their susceptibility, the IC50/LD50 for hydrogen peroxide was determined for both the *in vitro* and *in vivo* model systems used. In the CHO-K1 *in vitro* model, the IC50 of H_2_O_2_ was 140 μM (Figure 8) compared to the *G. mellonella in vivo* model that had an LD50 of 675 μM (Figure 9) for 20 μl of injected hydrogen peroxide. While the mode of exposure of the larvae/cells is not comparable, this nonetheless supports the much higher tolerance observed for *G. mellonella* larvae injected with biomolecules compared to the direct exposure of CHO-K1 cells to biomolecule-supplemented medium.

## DISCUSSION

Whilst eukaryotic cells have developed methods of dealing with oxidative stress induced biomolecule modification and have mechanisms to restore redox balance, detrimental effects due to excessive ROS do occur. The downstream biological effects of these structural changes remain to be investigated. To elucidate the mechanisms of actions and potential long-term effects of cold plasma, we chose four biomolecules involved in diverse biochemical pathways and a multitude of bioprocesses including; cellular respiration, intracellular transport, cell signaling or cell membrane structural components to analyse in this investigation. Plasma-induced chemical modifications of proteins, DNA, lipids and carbohydrates and associated structural modifications are dependent on gas composition, plasma discharge characteristics and liquid environment [22]. Results indicate that plasma induced chemical alterations to the biomolecular structure of these molecules has the potential to cause repercussions in cellular processes or to generate toxic/mutagenic metabolites.

The eukaryotic cell membrane is composed of two lipid monolayers embedded with proteins. The effects of cold plasma on cell membranes include transient pore formation through lipid peroxidation due to hydroxyl radicals [41]. H_2_O_2_ and O_3_ are indirectly involved in lipid peroxidation through the generation of hydroxyl radicals through the iron-catalysed Haber–Weiss reaction [42] and the lipid radical is able to propagate a chain reaction of lipid peroxidation of nearby lipids. The direct exposure to oxidants such as hydrogen peroxide or lipid hydroperoxides has been shown to directly induce apoptosis in various cell types [43]. Cholesterol is a monounsaturated fatty acid found in cell membranes and is prone to oxidation by oxygen free-radicals generating products such as hydroperoxides and oxysterols [40]. When cholesterol dissolved in deionised water was treated with plasma, significant cytotoxic effects were observed on CHO-K1 cells after just 5 min of plasma treatment. The H_2_O_2_ measurement of plasma treated cholesterol in H_2_O measured the highest of the biomolecules over 700 μM and can be correlated to the higher cytotoxic effects observed. However, as the IC50 indicates, H_2_O_2_ is not the only cytotoxic factor generated in the plasma treated solution. Despite the short-term cytotoxic effects observed for plasma treated cholesterol dissolved in H_2_O *in vitro*, the treated biomolecule was well tolerated by the *in vivo* model of *G. mellonella*. Plasma treated cholesterol also exhibited the lowest mutagenic potential of the selected biomolecules despite oxidation products of cholesterol such as epoxide demonstrating mutagenic potential *in vitro* [44, 45]. The short-term cytotoxicity observed in this study may be attributed to differences in the biochemical structures of the biomolecules and the aqueous environment they were treated in.

Arachidonic acid is an essential polyunsaturated fatty acid released during epithelial disruption and wound healing and is a crucial mediator of inflammation. Either directly or after enzymatic conversion to eiconsanoids, arachidonic acid modulates the function of various organs and systems including the digestive, renal, reproductive and immune systems [38]. The ability of arachidonic acid to induce cytotoxic effects, which were apoptotic in nature, in liver cells was predominantly attributed to lipid peroxidation and oxidative stress, where lipid peroxidation endproducts were detected and the exposure of cells supplemented with arachidonic acid to exogenous antioxidants provided protective effects [46]. The four double bonds make this molecule more susceptible to lipid peroxidation compared to the single double bond found in cholesterol and allow the lipid to react readily with molecular oxygen promoting oxidative stress [47] and may make it susceptible to lipid peroxidation by plasma reactive species. However, even after 10 min of plasma treatment when dissolved in H_2_O and used at 20% v/v in culture, cell growth showed very similar responses to those of cholesterol and remained just under 50%. Yet, arachidonic acid was the only biomolecule which showed pronounced cytotoxic effects at 10% v/v after plasma treatment in DMEM-F12. Arachidonic acid metabolism has been associated with the induction of genetic mutations by triggering hydroperoxide dependent oxidation products capable of inducing DNA damage and mutations [48]. Uncontrolled arachidonic acid lipid peroxidation and superoxide production may explain the cytotoxic and mutagenic potential of this bioactive molecule [39] and could play a role in the plasma-mediated effects observed here.

Serum albumin is the most abundant blood protein and acts as a circulating extracellular antioxidant. Its antioxidant properties arise from the flexible nature of the three domain design that enables the protein to adapt to a variety of ligands, including polyunsaturated fatty acids [38], long chain fatty acids (LCFA) and oxysterols. Albumin is important in the binding of the cationic ligands copper and iron preventing them from generating hydroxyl radials via the Fenton reaction with hydrogen peroxide. When BSA was dissolved in dH20, treated with plasma and cultured with CHO-K1 cells at 20% v/v, reduction of cell growth below 50% was observed after 5 min treatment time. BSA dissolved in DMEM-F12 and treated with plasma before being cultured with CHO-K1 cells at 10% v/v displayed no significant cytotoxic effects even at the prolonged treatment time of 10 min. The effects of plasma on protein structure are well documented. Unfolding and loss of activity in the model protein lysozyme was hypothesised to be due to chemical modifications of amino acids based on shifts in protein mass [49]. Oxidation of BSA and free methionine has been demonstrated using a capillary plasma jet [50] and investigations using a μAPPJ to treat BSA as a model protein indicated oxidation of sulfur-containing amino acids but no modification to cysteine involved in disulphide bonds [51]. Other studies using DBD plasma systems indicated that the thiol group of cysteine is modified by reactive oxygen and nitrogen species [52] and showed inactivation of proteins such as RNase by oxidation of sulfur-containing amino acids and over-oxidation of disulfide bonds [53].

Glucose was utilised in this study as a model carbohydrate and ubiquitous form of energy in cells. Plasma treated glucose exhibited the lowest cytotoxic potential under both treatment conditions but the highest mutagenic potential. H_2_O_2_ measurements were no different than the other treated biomolecules at 400 μM in H_2_O and 600 μM in DMEM-F12. During carbohydrate oxidation, the hydroxyl groups are oxidised to carbonyl groups and then carboxyl groups. Li et al found that treating sugars including glucose in solutions of water and PBS with DBD plasma caused the decomposition of these sugars into formic acid, glycolic acid, glyceric acid, tartaric acid and oxalic acid in time dependent concentrations and these effects were attributed to reactive oxygen species, primarily the hydroxyl radical [54].

In summary, a general increase in mutagenesis was observed over time and with longer plasma treatment times in all biomolecules. This was not unexpected as plasma technology has proven to be a powerful mutagenesis tool in microbial breeding in bacteria, fungi and microalgae, causing greater DNA damage and higher mutation rate than conventional mutagenesis methods [35, 36]. A possible limitation in this study was the ability to detect mutations in mammalian cells which varies depending on the locus examined using a single-copy gene whose inactivation by the mutagen causes a detectable phenotype, allowing small-scale detection of deletions, transitions or transversions.

All of the tested biomolecules were well-tolerated in the short-term by the *in vivo Galleria* model after single exposure via injection and importantly this suggests that higher organisms may possess sufficient mechanisms for detoxifying toxic components in the plasma treated liquids to prevent noticeable impacts on morbidity or mortality. However, the injection of this larvae model with lettuce broth treated with the same plasma system showed severe toxicity of 5 min treated product in another study [55]. The disparity between the short- and long-term effects and the influence of the aqueous milieu in this study highlights the need for more extensive investigations into the conformational changes on biomolecules post-plasma treatment and the effect that such intended or unintended changes would have on biological pathways.

The large-scale biomedical application of cold plasma or wider applications in food preservation or disinfection require adequate scientific research and technical data evaluating the overall safety considerations of cold plasma treatment. Cytotoxic and mutagenic responses of plasma treated biomolecules observed *in vitro* may not carry over to the *in vivo* model. This could be due to numerous reasons, including metabolic transformation. Even if a possible mutagen is produced, it may not reach the target organ or cells in high enough concentrations to cause genetic damage. Cells and especially more complex cellular structures and organisms have developed a range of mechanisms to remove defective molecules such as the degradation of damaged proteins through the proteasome. The longevity of alterations to the biomolecules and their purpose or elimination in a biological system need to be considered, where their persistence may not be long enough to cause systemic toxicity or long-term effects such as genotoxicity. The *in vivo* model presented in this study represents a short-term toxicity study while the HPRT assay is a long-term model of continuous and therefore excessive exposure to the plasma-treated substances. Both approaches can add insight to the overall question of plasma technology and applications safety. Our short-term *in vivo* tolerance results agree with a number of studies showing tissue tolerance to plasma and low *in vitro* toxic and mutagenic risks, suggesting that plasma reactive species in limited doses are safe. Adverse cyto- and genotoxic effects of high cumulative doses over long-term nonetheless indicate the need for further extensive studies to define the limits beyond which treatments could pose risk. *In vivo* models on mammals to examine possible mutagenic manifestations of direct plasma treatment or indirect plasma treatment including ingestion of plasma treated foods or liquids or application of plasma treated solutions should also be performed [56].

## MATERIALS AND METHODS

All chemicals were obtained from Sigma-Aldrich (Arklow, Ireland) unless specified otherwise.

### Plasma System

Cold plasma was generated using the previously described [57] high-voltage dielectric barrier discharge atmospheric cold plasma system, DIT-120, characterized in detail by Moiseev et al [58]. For the generation of plasma activated model solutions, samples were placed in Petri dishes inside a polypropylene container that operated as the dielectric barrier and the sample holder. The container was sealed in an air-tight film to ensure species retention. Plasma was generated by sine-wave excitation at 80 kV RMS and 50 Hz (ac frequency) in air and the distance between electrodes was kept constant at 3cm for all experiments. Model solutions were subjected to 24-h post treatment storage time within the sealed container before opening.

### Preparation of Biomolecule Solutions

All biomolecules were dissolved within physiological ranges (BSA 530 mmol/l; glucose 4 mmol/l; cholesterol 5 mmol/l; arachidonic acid 10 μmol/l). For the short term *in vivo* and *in vitro* cytotoxicity study, biomolecules were dissolved in deionised water prior to plasma treatment. For the long term mutagenicity study, biomolecules were dissolved in DMEM-F12 prior to plasma treatment. All solutions were filter sterilised with a 0.2 μm syringe filter before and after plasma treatment.

### Cell Culture

The Chinese hamster cell line CHO-K1 was used for the cytotoxicity and mutagenicity studies. CHO-K1 cells were cultivated in DMEM/F12 with 2 mM L-glutamine and 10% foetal bovine serum [24]. Cells were grown at 37°C and 5% CO_2_ in a humidified incubator. Cells were detached using trypsin/EDTA and cell concentrations and viability were assessed using trypan blue exclusion assay. Cell viability was assessed by crystal violet staining: trypsinized cells seeded at 2.5 × 10^4^ cells/ml were left to re-adhere and grow for 3 days. Culture supernatant was removed and cells were fixed with 70% methanol for 1 min followed by staining with 0.2% crystal violet solution for 10 min. Cells were washed with water and allowed to air-dry. Adherent crystal violet was dissolved with 10% acetic acid and the absorbance was measured at 600 nm on a spectrophotometric microplate reader (Biotek, Winooski, United States). Cell growth was expressed as a percentage of control cells.

### HPRT Assay

The hypoxanthine phosphoribosyl transferase (HPRT) assay was employed to detect the potential of plasma treated biomolecules to induce mutations in the CHO-K1 cell line. Cells were cultured in T25 flasks or 6-well plates in DMEM/F12 medium supplemented with 10% FBS and 10% biomolecule solution, treated at 80 kV RMS for 0, 1, 5, and 10 min with 24 h post-treatment storage time. Cells were passaged every 3–4 days through trypsinisation and reseeded at 2.5 × 10^4^ cells/ml into fresh 6-well plates. Once a week during reseeding, cells were also plated at 1 × 10^4^ cells/ml in 60mm round dishes with DMEM/F12 10% FBS and 10 μg/ml 6-thioguanine (6-TG) as a selection agent. Colony formation was determined after 10–14 days of incubation at 37°C and 5% CO_2_ by staining with crystal violet. Plates were recorded as HPRT+ or HPRT-based on the existence of colonies. CHO-K1 cells were cultured with plasma treated solutions in three independent models and 6-TG plates were set up in triplicate for each replicate. Data is presented as cumulative data in the form of percentage positive plates of total plates assessed at each time point. Control plates were negative for colony formation at the start of the 40 days exposure; ethyl methanesulfate (EMS) was used as a positive control to induce colony formation.

### Insect Larvae

Sixth-star *G. mellonella* larvae were obtained commercially from livefoods.direct.co.uk and stored in wood shavings at 15°C prior to use. Dead larvae and those showing signs of melanisation were discarded. Three groups of ten randomly-selected larvae, each weighing 0.2–0.3 g were used for each treatment.

### *Galleria mellonella* Intra-haemocoel Inoculation

The *G. mellonella* haemocoel was injected with 20 μl of selected biomolecular solution using a 0.3 ml Terumo^®^ Myjector^®^ U-100 insulin syringe through the base of the last proleg. Three control groups of ten larvae were injected with sterile deionised H_2_O. Larvae were incubated at 30°C for 24 h. Larvae were assessed visually for viability 24 h after injection with plasma treated biomolecule solutions and percentage survival was noted. Larvae were considered dead if they were unmoving, failing to reorient themselves if placed on their backs or failed to respond to stimuli [59].

### Harvesting of Insect Haemocytes

After 24 h incubation at 30°C, haemolymph was drained from five larvae in each group by piercing the anterior region and draining into chilled 1.5 ml microfuge tubes, which were kept on ice to prevent melanisation of the haemolymph. All samples were diluted by adding 100 μl haemolymph to 900 μl ice-cold PBS and for each extract concentration, haemocytes were enumerated microscopically using a haemocytometer, and compared to haemolymph samples of control larvae which were injected with 20 μl of sterile H_2_O.

### Colorimetric Determination of Peroxide Concentration

Concentrations of peroxide in plasma treated solutions were ascertained via spectrophotometrically measuring the oxidation of potassium iodide to iodine at 390 nm. 50 μl of phosphate buffer and 100 μl of 1 M KI solution were added to 50 μl of plasma treated biomolecule solution and incubated at room temperature for 30 min. Absorbance was read at 390 nm and a standard curve of known hydrogen peroxide concentrations was generated with each plate to correlate absorbances with peroxide concentrations.

### Statistical Analysis

Experiments were performed in triplicate and results are presented as means with standard deviations and statistical analysis where applicable was performed by analysis of variance (ANOVA) using GraphPad Prism (GraphPad Software Inc., La Jolla, United States). Results obtained from the HPRT assay are presented as cumulative data of experiments performed in triplicate with three plates per replicate.

## Supplementary Material

Table S1

## Data Availability

The raw data supporting the conclusions of this article will be made available by the authors, without undue reservation.
