# Supplementary material for: Biomolecules as Model Indicators of In Vitro and In Vivo Cold Plasma Safety
Source: Front Phys. Author manuscript; Available in PMC 2023 Apr 27. (PMC10136044; doi:10.3389/fphy.2020.613046)
Supplement: Table S1 [file NIHMS1875081-supplement-Table_S1.docx]

Table S1: Occurrence of HPRT+ colonies in cultures supplemented with plasma treated BSA, arachidonic acid, glucose or cholesterol exposed to DBD-ACP for 0, 1, 5 or 10 min over a 34-day cultivation period. *minus* indicates no colonies were observed; *plus* indicates one or more colonies were observed; nd: not determined; na: not assessable.

| **Bovine Serum Albumin** | Days in Culture | | | | | | |
| --- | --- | --- | --- | --- | --- | --- | --- |
|  |  | 0 | 6 | 13 | 20 | 27 | 34 |
| Control  Untreated | A | nd | -/-/- | -/-/- | -/-/- | -/-/- | -/-/- |
|  | B | nd | -/-/- | -/-/- | -/-/- | -/-/- | -/-/- |
|  | C | nd | -/-/- | -/-/- | -/-/- | -/-/- | -/-/na |
| 1 min | A | nd | -/-/- | -/-/- | -/-/- | -/-/- | -/-/- |
|  | B | nd | -/-/- | -/-/- | -/-/- | -/-/- | -/-/- |
|  | C | nd | +/-/- | -/-/- | -/-/- | -/-/- | -/-/- |
| 5 min | A | nd | -/-/+ | -/-/- | -/-/- | -/-/- | -/na/- |
|  | B | nd | +/-/- | -/-/- | -/-/- | -/-/- | -/-/- |
|  | C | nd | -/-/- | -/-/- | -/-/- | +/-/+ | -/-/na |
| 10 min | A | nd | -/-/- | -/+/- | +/-/+ | +/+/+ | na/-/- |
|  | B | nd | +/-/- | +/+/- | -/+/- | -/+/- | -/-/- |
|  | C | nd | -/-/- | -/-/- | -/-/- | -/+/- | -/-/- |
| **Arachidonic Acid** | Days in Culture | | | | | | |
|  |  | 0 | 6 | 13 | 20 | 27 | 34 |
| Control  Untreated | A | nd | -/-/- | -/-/- | -/-/- | -/-/- | -/-/- |
|  | B | nd | +/-/+ | -/-/- | -/-/- | -/-/- | -/-/- |
|  | C | nd | -/-/- | -/-/- | -/-/- | -/-/- | -/-/- |
| 1 min | A | nd | -/-/- | -/-/- | -/-/- | -/-/- | -/-/- |
|  | B | nd | -/+/+ | -/-/- | +/-/+ | -/-/- | -/-/- |
|  | C | nd | +/-/+ | -/-/- | -/-/- | -/-/- | -/-/- |
| 5 min | A | nd | -/-/- | -/-/+ | +/+/+ | -/-/- | na/+/- |
|  | B | nd | -/-/- | -/-/- | -/-/- | +/-/+ | -/-/na |
|  | C | nd | -/-/- | -/-/- | -/-/- | -/-/- | na/-/- |
| 10 min | A | nd | -/-/- | -/-/- | +/-/- | -/-/- | +/+/- |
|  | B | nd | -/-/- | -/-/- | -/+/- | -/-/+ | -/na/na |
|  | C | nd | -/-/- | -/-/- | +/-/+ | +/+/+ | na/na/na |
| **Glucose** | Days in Culture | | | | | | |
|  |  | 0 | 6 | 13 | 20 | 27 | 34 |
| Control  Untreated | A | nd | -/-/- | -/-/- | -/-/- | -/-/+ | -/-/- |
|  | B | nd | +/-/- | -/-/- | +/-/- | -/-/- | -/-/- |
|  | C | nd | -/-/- | -/-/- | -/-/- | -/+/- | -/-/- |
| 1 min | A | nd | -/-/- | -/-/- | -/-/- | -/-/+ | -/-/- |
|  | B | nd | -/-/- | -/-/- | -/-/- | -/-/- | -/-/- |
|  | C | nd | +/-/+ | -/-/- | -/-/- | -/-/- | -/-/na |
| 5 min | A | nd | -/-/- | -/-/- | -/-/- | -/-/- | -/+/- |
|  | B | nd | +/-/- | -/-/- | -/-/- | -/-/- | -/-/- |
|  | C | nd | -/-/- | -/-/+ | -/-/- | -/-/- | -/-/- |
| 10 min | A | nd | -/-/+ | -/-/- | +/+/+ | +/+/+ | na/-/+ |
|  | B | nd | -/-/- | -/+/- | +/+/- | +/+/+ | -/-/- |
|  | C | nd | +/+/- | -/-/- | -/-/- | -/-/+ | -/-/- |
| **Cholesterol** | Days in Culture | | | | | | |
|  |  | 0 | 6 | 13 | 20 | 27 | 34 |
| Control  Untreated | A | -/-/- | -/-/- | -/-/- | -/-/- | -/-/- | -/-/- |
|  | B | -/-/- | -/-/- | -/-/- | -/-/- | -/-/- | -/-/- |
|  | C | -/-/- | -/-/- | -/-/- | -/-/- | -/-/- | -/-/- |
| 1 min | A | nd | -/-/- | -/-/- | -/-/- | -/-/- | -/-/- |
|  | B | nd | -/-/- | -/-/- | +/+/- | -/-/- | -/-/- |
|  | C | nd | -/-/- | -/-/- | -/-/- | -/-/- | -/-/- |
| 5 min | A | nd | -/-/- | -/-/- | +/-/- | -/-/- | -/-/- |
|  | B | nd | -/-/- | -/-/- | -/-/- | -/-/- | -/-/- |
|  | C | nd | -/-/- | -/-/- | -/-/- | -/-/- | -/-/- |
| 10 min | A | nd | -/-/- | -/-/- | -/-/- | -/-/- | -/-/- |
|  | B | nd | -/-/- | -/-/- | -/-/- | -/-/- | -/-/- |
|  | C | nd | -/-/- | -/-/- | -/+/- | +/+/- | -/+/- |
